# Supplementary material for: Quantitative receptor model for responses that are left- or right-shifted versus occupancy (are more or less concentration sensitive): the SABRE approach
Source: Front Pharmacol. 2023 Dec 15;14:1274065. doi: 10.3389/fphar.2023.1274065 (PMC10755021; doi:10.3389/fphar.2023.1274065)
Supplement: Supplementary file 1 [file DataSheet1.pdf]

SUPPLEMENTARY INFORMATION

Quantitative Receptor Model for Responses That Are Left- or Right-Shifted Versus Occupancy (Are More or Less Concentration Sensitive): The SABRE Approach

Peter Buchwald[*]

Department of Molecular and Cellular Pharmacology and Diabetes Research Institute, Miller School of Medicine, University of Miami, Miami, FL, USA

- Supplementary Tables
  - Table S1. Detailed parameters from fitting shown in Figure 2.
  - Table S2. Detailed parameters from fitting shown in Figure 6.
- Supplementary Figures
  - Figure S1. Illustration of a right-shifted response that needs ν = 2 independent receptors occupied to trigger it.

Supplementary Tables

Supplementary Table S1. Parameters and quality of fit descriptors for data shown in Figure 2 [experimental data from (Ruffolo et al., 1979)]. All fittings done in GraphPad Prism.

**Parameter**	**Phenyl-ephrine**	**Oxy-metazoline**	**Naphazoline**	**Clonidine**	**Tolazoline**	**Tena-phtoxaline**	**Tetra-hydrozoline**
**A**. Experimental data [from (Ruffolo et al., 1979)][a]							
log K_d	-6.46	-6.36	-8.23	-7.66	-6.69	-7.53	-7.26
log EC_{50}	-7.55	-6.77	-8.20	-7.60	-6.64	-7.51	-7.16
$E_{max,L}$ ($f_{resp,max}$)	1.000	0.730	0.480	0.330	0.100	0.270	0.070
κ (fold vs occup.)	12.30	2.57	0.94	0.88	0.90	0.95	0.79
**B**. Fit with standard E_{max} (eq. 2)							
log EC_{50}	-7.55	-6.77	-8.20	-7.60	-6.64	-7.45	-7.16
e_{max}	0.996	0.729	0.480	0.332	0.100	0.276	0.068
r^2	0.996	0.998	0.997	0.994	0.949	0.977	0.978
SSE	56.25	10.50	4.26	7.52	2.95	9.41	0.33
**C**. Fit with SABRE (eq. 4 with experimental K_d)							
log K_d (from exp.)	-6.46	-6.36	-8.23	-7.66	-6.69	-7.53	-7.26
γ	11.63 ± 1.83						
ε	1.000	0.177	0.065	0.038	0.009	0.029	0.006
↓[b]							
log EC_{50} (K_{obs})	-7.53	-6.82	-8.46	-7.81	-6.73	-7.65	-7.29
e_{max} ($f_{resp,max}$)	1.000	0.715	0.447	0.313	0.096	0.261	0.065
κ	11.63	2.88	1.69	1.40	1.10	1.31	1.06
r^2	0.996	0.997	0.951	0.974	0.943	0.941	0.959
SSE	58.54	16.88	75.88	31.64	3.304	24.27	0.65
**D**. Fit with SABRE for f_{resp} vs. f_{occup} directly (eq. 17)							
γ	11.63 ± 1.83						
ε	1.000	0.177	0.065	0.038	0.009	0.029	0.006
r^2	0.996	0.997	0.951	0.974	0.943	0.941	0.959
SSE	58.54	16.88	75.88	31.64	3.304	24.27	0.65

[a] Experimental data – average of log K_A and log K_B from Table 3 and 4 in (Ruffolo et al., 1979).; κ (fold shifts vs occupancy) calculated from the K_d/EC_{50} values. [b] Derived values for the present model (using eqs. 6, 7, and 17). Quality of fit descriptors included: r^2, correlation coefficient; SSE, sum of squared errors.

Supplementary Table S2. Parameters and quality of fit descriptors for data shown in Figure 6 [experimental data from (Pedersen et al., 2019)]. All fittings done in GraphPad Prism.

Parameter	Loperamide	DAMGO	Morphine	Buprenorphine	Oliceridine (R)-TRV130	(S)-TRV130
**A**. Experimental data [from (Pedersen et al., 2019)]						
log K_d[a]	-7.64	-7.34	-7.02	-8.37	-7.29	-5.73
log $EC_{50,Gprt}$	-9.20	-8.61	-8.17	-9.26	-8.49	-6.53
$E_{max,Gprt}$ ($f_{resp,max}$, %)	98	99	98	84	82	85
log $ED_{50,\beta Arr}$	-6.94	-6.10	-6.00			
$E_{max,\beta Arr}$ ($f_{resp,max}$, %)	51	99	25			
**B**. Fit with standard E_{max} (eq. 2)						
log $EC_{50,\ Gprt}$	-8.98	-8.62	-8.09	-8.66	-8.51	-6.53
$e_{max,Gprt}$	105.4	96.9	98.1	83.8	84.3	86.6
log $EC_{50,\beta Arr}$	-6.89	-6.12	-6.10			
$e_{max,\beta Arr}$	52.9	97.9	23.1			
**C**. Fit with SABRE (eq. 4 with experimental K_d)						
log K_d (from exp.)[a]	-7.64	-7.34	-7.02	-8.37	-7.29	-5.73
γ_{Gprt}	19.88					
$\gamma_{\beta Arr}$	0.067					
ε	0.950	0.998	0.792	0.141	0.382	0.258
↓[b]						
log $EC_{50,\ Gprt}$	-8.98	-8.70	-8.29	-8.98	-8.26	-6.55
$e_{max,Gprt}$	99.8	100.0	98.9	79.1	93.4	88.9
log $EC_{50,\beta Arr}$	-6.69	-6.17	-6.43	-8.31	-7.10	-5.61
$e_{max,\beta Arr}$	55.1	96.7	19.9	1.1	3.9	2.2

[a] Experimental data from (Pedersen et al., 2019). [b] Derived values for the present model (using eqs. 6 and 7).

Supplementary Figures

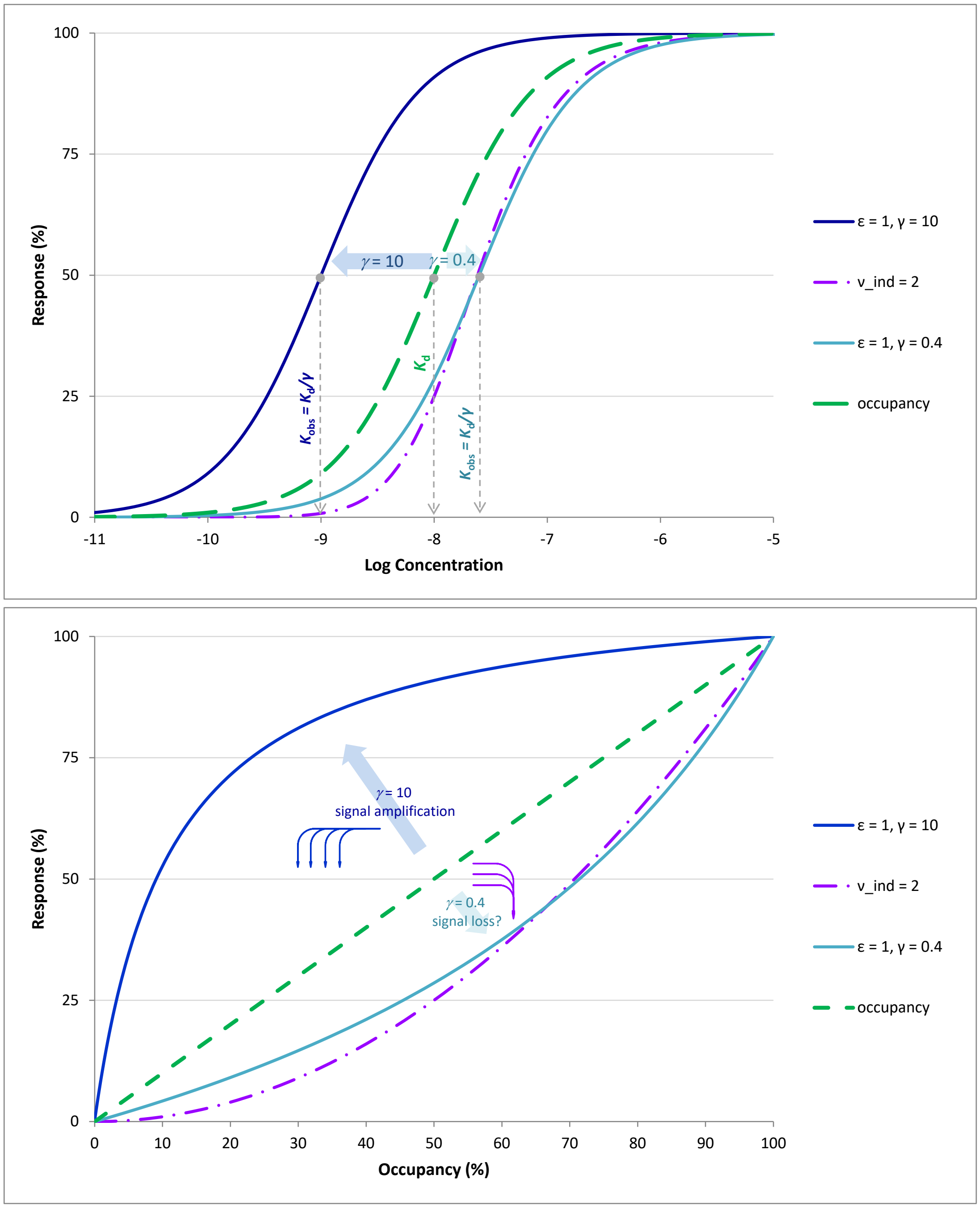

Figure S1. Illustration of a right-shifted response (dashed purple line) caused by the assumption that $\nu = 2$ independent receptors within the same system need to be occupied to trigger the response, which result in $f_{resp} \propto (f_{occup})^{\nu}$. Responses are shown as a function of log concentration (top) or fractional occupancy (bottom).
